# A first insight into genetic diversity of *Mycobacterium bovis* isolated from extrapulmonary tuberculosis patients in South Tunisia assessed by spoligotyping and MIRU VNTR

**DOI:** 10.1371/journal.pntd.0007707

**Published:** 2019-09-18

**Authors:** Mariam Siala, Cécile Cassan, Salma Smaoui, Sana Kammoun, Chema Marouane, Sylvain Godreuil, Salma Hachicha, Emna Mhiri, Leila Slim, Dhikrayet Gamara, Férièle Messadi-Akrout, Anne-Laure Bañuls

**Affiliations:** 1 Department of Biology, Preparatory Institute for Engineering Studies, Sfax, University of Sfax-Tunisia; 2 Department of Life Sciences, Research Laboratory of Environmental Toxicology-Microbiology and Health (LR17ES06), Faculty of Sciences, Sfax, University of Sfax-Tunisia; 3 MIVEGEC, UMR IRD–CNRS—Université de Montpellier, Montpellier, France; 4 Department of Mycobacteriology, Regional hygiene care laboratory, Hedi-Chaker University Hospital, Sfax, Tunisia; 5 Department of Biology B, Faculty of pharmacy, Monastir, University of Monastir, Monastir, Tunisia; 6 Laboratoire de Bactériologie, CHU de Montpellier, France; 7 Department of Microbiology, National Reference Laboratory of Mycobacteria, Research Unit (UR12SP18), A. Mami University Hospital of Pneumology, Ariana, Tunisia; 8 Basic Health Care Management, Ministry of Health, Tunis, Tunisia; University of California, Davis, UNITED STATES

## Abstract

**Introduction:**

In Tunisia, almost 77% of clinically and bacteriologically diagnosed cases of extrapulmonary tuberculosis (EPTB) are zoonotic TB, caused by *M*. *bovis*. Although several studies have analyzed bovine TB in cattle in Tunisia, no study has evaluated the risk of transmission to humans in such an endemic country. We aimed to study the genetic diversity of *M*. *bovis* human isolates, to ascertain the causes of human EPTB infection by *M*. *bovis* and to investigate the distribution and population structure of this species in Tunisia.

**Materials and methods:**

A total of 110 *M*. *bovis* isolates taken from patients with confirmed EPTB were characterized by spoligotyping and MIRU-VNTR typing methods.

**Results:**

Among the 15 spoligotypes detected in our study, 6 (SB0120, SB0121, SB2025, SB1200, SB1003 and SB0134) were the most prevalent (83.5%) of which SB0120, SB0121 and SB2025 were the most prevailing. MIRU-VNTR typing method showed a high genotypic and genetic diversity. The genetic differentiation based on MIRU-VNTR was significant between populations from South East (Tataouine, Medenine) and Central West (Gafsa, Sidi Bouzid, Kasserine) regions. Of note, 13/15 (86.7%) spoligotypes detected in our study were previously identified in cattle in Tunisia with different frequencies suggesting a peculiar ability of some genotypes to infect humans. Using combined spoligotyping and MIRU-VNTR method, a high clustering rate of 43.9% was obtained. Our results underlined that human EPTB due to *M*. *bovis* was more commonly found in female gender and in young patients. Most of our patients, 66.4% (73/110) were raw milk or derivatives consumers, whereas 30.9% (34/110) patients would have contracted EPTB through contact with livestock. The findings suggest that the transmission of Zoonotic TB caused by *M*. *bovis* to humans mainly occurred by oral route through raw milk or derivatives.

**Conclusion:**

Our study showed the urgent need of a better veterinary control with the implementation of effective and comprehensive strategies in order to reach a good protection of animals as well as human health.

## Author summary

In South Tunisia, the prevalence of bovine TB is high with *Mycobacterium bovis* as causative agent and cattle as reservoir of the bacteria. However as previously mentioned in several studies, *M*. *bovis* is also responsible for human extrapulmonary tuberculosis (EPTB) cases in South Tunisia. Despite the veterinary and medical problems, *M*. *bovis* is still little studied. In this context, this work aimed to study the molecular epidemiology of *M*. *bovis* in EPTB patients in south Tunisia in order to determine the main risk factors of transmission. Our results underlined that SB0120, SB0121 and SB2025, previously described in cattle in Tunisia, represent the predominant genotypes. The findings highlighted that human EPTB caused by *M*. *bovis* mainly occurred through the consumption of raw milk or derivatives. These data demonstrate the urgent need to implement strategies for preventing and controlling zoonotic TB.

## Introduction

In developing countries, *Mycobacterium bovis*, the agent of bovine tuberculosis (bTB) represents a threat for livestock and human health [1, 2]. Humans can acquire *M*. *bovis* either by aerogenous route due to a close contact with infected animals or by consuming unpasteurized dairy products [1]. According to the World Health Organization (WHO), among the estimated 10 million new cases of tuberculosis (TB) in 2017, almost 20% were extrapulmonary tuberculosis (EPTB) [3]. Furthermore, in the region with high bTB prevalence, *M*. *bovis* would frequently be associated with extrapulmonary disease in humans [1, 3, 4]. In developing countries, the exact percentage of *M*. *bovis* in human TB cases is generally underestimated, since the species diagnosis is rarely performed, especially in EPTB cases [1, 3, 4].

Human TB (30/100.000) is endemic in Tunisia with high frequency of EPTB cases (56.9%) despite the low rate of HIV infection [5]. Recently, Ghariani *et al* have reported high prevalence of *M*. *bovis* (76%) in lymphadenitis TB (LTB) cases in North Tunisia by conventional methods [6]. In South Tunisia, Siala *et al* have detected *M*. *bovis* by qPCR in almost 77% of extrapulmonary samples taken from patients with EPTB [7]. In addition, LTB was estimated to be 50% of all EPTB cases of which cervical localization was the most frequent (70% to 90%) [5, 8]. Djemal *et al*, have demonstrated that *M*. *bovis* is still spreading in Tunisia causing bTB with a frequency of 64.4% in 2014–2015, whereas Lamine-khmiri *et al*, reported a frequency of 35% in 2013 [9, 10]. Consequently, the risk of contracting EPTB is still high since the control measures for herd, livestock and unpasteurized dairy products as well as milk are consistently declining [1, 7]. Nevertheless, no study evaluated the risk of transmission of zoonotic TB due to *M*. *bovis* to humans in such an endemic country. Furthermore, the genetic background of *M*. *bovis* causing human EPTB is still not known in Tunisia. The availability of such information is critically important in order to identify the source, the route of infection and thus to control the disease. The main goal of this work was to genetically characterize the *M*. *bovis* population involved in human EPTB in order to improve our understanding of the bTB epidemiology and *M*. *bovis* spread in Tunisia. Spoligotyping and mycobacterial interspersed repetitive units-variable number of tandem repeats (MIRU-VNTR) analysis were used, since the combination of these two genetic markers is known to be a powerful tool for the molecular epidemiology study of *M*. *bovis* [11,12]. We finally compared *M*. *bovis* data obtained from humans with data from Tunisian cattle [9, 10].

## Materials and methods

### Ethics statement

Approval for usage of *M*. *bovis* isolates, demographic, epidemiological and clinical data for our study was obtained by the Ethics Committee of Hedi Chaker Hospital-Sfax-Tunisia

### Patients

The study was prospectively conducted over a 32-month period (January 2013 to September 2015) by the regional hygiene care mycobacteriology laboratory of the Department of Microbiology of the Hedi-Chaker University Hospital in Sfax (South Tunisia). During this period, all patients with a positive diagnosis of extrapulmonary tuberculosis (EPTB), microbiologically confirmed and attributed to *M*. *bovis*, were included in the study. All data were anonymized. The laboratory provided routine mycobacterial diagnostic tests for TB specimens obtained during consultation or hospitalization of suspected TB patients in Hedi-Chaker University Hospital.

In total, 110 patients were included in this study. One hundred and six patients were from 7 Tunisian governorates localized in East Central Tunisia (Sfax (n = 23), Gabes (n = 21)), West Central Tunisia (Gafsa (n = 13), Sidi bouzid (n = 7), Kasserine (n = 4)) and South East (Tataouine (n = 23), Medenine (n = 15)) of Southern Tunisia regions. Four patients were from Libya. The other demographic and clinical characteristics that were collected include sex, age, clinical site of TB, patient origin, past history of TB, raw milk consumption, contact with livestock and BCG vaccination status (Table 1 and S1 Table).

**Table 1:** Demographic and clinical characteristics of the 106 Tunisian extrapulmonary tuberculosis patients and the 4 isolates from Libyan patients.

Variants	N°. (%) of patients
**Sex**	
Male	33 (30)
Female	77 (70)
**Age, years**	
0–4 years	8 (7.3%)
5–14 years	13 (11.8%)
15–59 years	84 (76.4%)
≥ 60 years	5 (4.5%)
**Main site of EPTB**	
Lymphatic	100 (90.9)
others	10 (9.1)
**Patient origin**	
Central East region (Sfax, Gabes)	44 (40)
Central West region (Gafsa, Sidi Bouzid, Kasserine)	24 (21.8)
South East region (Tataouine, Medenine)	38 (34.5)
Libya	4 (3.6%)
**Raw milk consumption**	
Yes	73 (66.4)
No	30 (27.3)
No data	7 (6.4)
**Contact with livestock**	
Yes	34 (30.9)
No	53 (48.2)
No data	23 (20.9)
**TB history**	
Yes	6 (5.4)
No	60 (54.5)
No data	44 (40)
**BCG vaccination**	
Yes	62 (56.4)
No	3 (2.7)
No data	45 (40.9)

### Isolation and identification of mycobacteria

Specimens from various extrapulmonary sites were collected: lymph nodes (n = 100), osteoarticular, intestinal, meningeal, peritoneal and pleural samples (n = 10). Solid specimens were homogenized with pestle and mortar. All specimens were directly inoculated on Lowenstein–Jensen (LJ) and Coletsos slants. After decontamination using the standard N-acetyl- L-Cysteine sodium hydroxide method, the samples were centrifuged at 3000 rpm for 15 min. The pellets were then inoculated on Lowenstein–Jensen (LJ), Coletsos media, and in liquid media Mycobacterial Growth Indicator Tube 960 (MGIT 960, Becton Dickinson Biosciences, Sparks, MD, USA). The tubes were incubated at 37°C for 42 days and the cultures were incubated on solid media at 37°C for up to 12 weeks.

The isolates were identified as mycobacteria and *M*. *bovis* species by Ziehl-Neelsen (ZN) staining for Acid Fast Bacilli (AFB), morphological and biochemical criteria including niacin test, nitrate reductase test, and susceptibility for certain inhibitors such as thiophene-2-carboxylic acid hydrazide (TCH), pyrazinamid and p-nitrobenzoic acid (PNB) [13]. Strain identification was confirmed by the commercial multiplex line probe assay, GenoTypeMTBC (Hain Lifescience GmbH, Nehren, Germany) [14].

### Genotyping

DNA extraction from mycobacteria isolates and high-throughput spoligotyping on Luminex 200 (Luminex Corp., TX) were performed as previously described [15, 16, 17, 18]. The obtained data were compared with those of the international database (www.mbovis.org). The isolates were also genotyped by PCR amplification of 8 Mycobacterial Interspersed Repetitive Units-Variable Number Tandem Repeats (MIRU-VNTR) loci: ETR-A (VNTR 2165), ETR-B (VNTR 2461), QUB 11a (VNTR 2163a), QUB 11b (VNTR 2163b), QUB 26 (VNTR 4052), QUB 3232 (VNTR 3232), ETR-C (VNTR 0577) and MIRU 4, ETR-D (VNTR 580) as described previously [19, 20, 21]. These eight loci were selected according to the recommendation of the European Union Reference Laboratory (EURL) [21]. The results were combined into 8-digit allelic profiles for each isolate [19]. The spoligotyping and MIRU-VNTR data obtained from *M*. *bovis* isolated from Tunisian cattle were used for comparison [9, 10].

### Genetic diversity and population structure analyses

Several diversity indices, including the genotypic diversity (*G*d = the number of different genotypes divided by the total number of samples using the combination of MIRU-VNTR and Spoligotyping data), the allelic diversity per locus and the mean genetic diversity (*H*s) were calculated. The population structure was explored by estimating the *F*_st_ (index of genetic differentiation between samples) value (0 = no differentiation and 1 = fixation of alternative alleles). The allelic diversity, the *H*s and the *F*_st_ were calculated using F-STAT, version 2.9.3 using the 8 MIRU-VNTR loci data [22]. The discriminatory power of each locus and of the typing method was estimated using the Hunter-Gaston discriminatory Index (HGDI) according to the previously described formula [23].

### Phenetic tree and statistical analyses

Neighbor-joining tree was constructed based on Cavalli-Sforza and Edwards distance methods, using Populations 1.2.30 (Olivier Langella, CNRS UPR9034, France) [24] and MEGA6 [25] software. The topology robustness was estimated by performing bootstrap analysis with 1000 replicates. Treedyn and Inkscape were used for tree visualization and annotation [26]. The clustering rate was calculated using the N-1 method formula as described previously [27] to estimate the proportion of TB potentially attributable to recent transmission.

Univariate analysis was performed to test the association of risk factors with clustering (proportion of isolates in clusters versus non-clustered ones). Statistical analyses (chi-square, Fisher’s exact 2-tailed and student test) were performed using SPSS 16.0 statistical software (SPSS Inc., Chicago, IL). Differences were considered significant at values of *P* ≤ 0.05.

## Results

### Sociodemographic and clinical data

The sociodemographic and clinical data linked to the EPTB human isolates are shown in Table 1 and S1 Table. For some patients, the epidemiological data could not be collected as mentioned in the tables.

LTB was the most common form (n = 100/110, (90.9%)) among the patients with *M*. *bovis* culture confirmed-EPTB, followed by osteoarticular (n = 4), pleural (n = 3), meningeal (n = 1), peritoneal (n = 1) and intestinal (n = 1) forms. Two out of the 110 *M*. *bovis* isolates under study were resistant to streptomycin and one to rifampicin and ethambutol.

The age of the patients ranged between 1 and 72 years, with a mean age of 30.36 ± 17.07 years (Table 1). The majority of the patients ranged between 15–59 years (n = 84, (76.4%)) with 49% of cases between 20 and 40 years old. The male/female ratio in the study population was 0.43. Age distribution differed significantly between men and women (mean age 24.21 ±20.03 years versus 33 years ±15.02; *P* = 0.013, respectively).

The majority of isolates were collected from Tunisian patients who originated from the Central Eastern (40%) and South Eastern Tunisia (34.5%), with the highest percentages in Tataouine (n = 23, 21%), Sfax (n = 23, 21%) and Gabes (n = 21, 19.1%) governorates. Four patients (3.6%) were from Libya (see Table 1, and S1 Table). A high proportion of patients (n = 73/110, (66.4%)) used to consume raw milk or derivatives with a significant increase observed in patients between 15–59 years (*p* = 0.04). TB history was recorded only for 6 patients (5.4%). None of them was older than 15 years (*p* = 0.02). About half of the patients received BCG vaccination and 34 (30.9%) had contact with livestock or other animals (Table 1).

Patients in contact with livestock (14/31 (45.2%)) and consuming raw milk (21/73 (28.8%)) were significantly more represented in the Central West region including Gafsa, Sidi Bouzid and Kasserine, than in the other regions (*p* = 0.004 and *p* = 0.039, respectively) (Table 1). Of note, 100% of patients from the Central West region had LTB. Isolates from Libyan patients were excluded in this latter statistical analysis.

### Genetic characterization of human *M*. *bovis* isolates

#### Spoligotyping analysis

Among the 110 human isolates, 109 revealed 15 spoligotypes and one did not give any spoligotype pattern (S1 Table). All the spoligotypes were already reported in the http://www.Mbovis.org database. From the 109 fully characterized isolates, 105 (96.3%) were classified as *M*. *bovis* bearing 13/15 (86.7%) of the detected spoligotypes (SB0120, SB0121, SB2025, SB1200, SB1003, SB0134, SB0871, SB0162, SB0133, SB1148, SB0119, SB1346, SB0828*)*. The two other spoligotypes (SB0866 and SB2024), represented by four isolates, were identified as *M*. *caprae*. Among the 15 spoligotypes found in this study, six patterns (SB0120, SB0121,SB2025, SB1200, SB1003 and SB0134) were the most detected representing 83.5% of all the isolates under study of which 3 were the most prevalent (SB0120, SB0121 and SB2025) (S2 Table).

**Fig 1:**
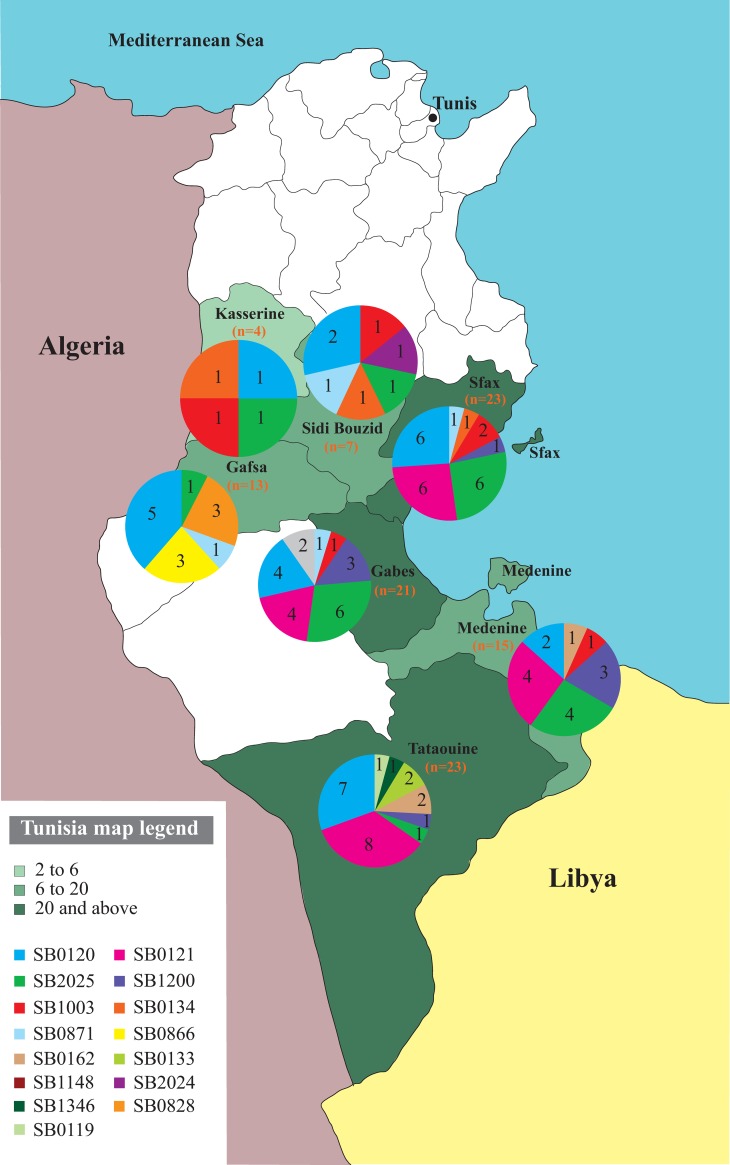
Map of Tunisia showing the localization of spoligotypes of *M*. *bovis* isolated from EPTB patients identified in 7 distinct governorates (n = 105/110 isolates, one strain from Gabes did not give any SB profile and the 4 isolates from Libyan patients were not presented). The map of Tunisia was created specifically for the manuscript using the GPL licenced software Epi Info 7.2.

Fig 1 shows the geographical distribution of the different spoligotypes. The governorates of Sfax, Gabes, and Tataouine revealed the highest genetic diversity with 8, 7 and 7 different spoligotypes, respectively (Fig 1).

The most frequent spoligotype was SB0120 usually referred to as ‘‘BCG-like” including 29/109 isolates (26.6%). SB0120 was reported in all the 7 Tunisian governorates under study (Fig 1) and in the 3 regions with almost the same proportion: 10/29 (34.5%) isolates in Central East Tunisia (i.e Sfax (n = 6), Gabes (n = 4)); 8/29 (27.6%) in Central West Tunisia (i.e Gafsa (n = 5), Sidi Bouzid (n = 2), Kasserine (n = 1)) and 9/29 (31%) in South East Tunisia (i.e Tataouine (n = 7), Medenine (n = 2)) (Fig 1, Table 1 and S1 Table). SB0120 was also observed in 2 isolates from the Libyan patients (S1 Table).

The other spoligotypes were detected differently between the regions and governorates as detailed in Fig 1 and S1 and S2 Tables. For the two last Libyan isolates, one belonged to SB1003 and the second to SB0134 (S1 Table).

Considering *M*. *caprae*, three out of the four isolates were grouped in one spoligotype (SB0866) and the remaining belonged to SB2024 (S1 Table). The *M*. *caprae* SB2024 pattern was found in Sidi Bouzid governorate and SB0866 was detected only in Gafsa governorate (Fig 1).

#### MIRU-VNTR analysis

The MIRU-VNTR analysis of the 110 isolates was more discriminatory as 60 profiles (P1-P60) were identified. P48 and P17, for which the locus ETR-A (sample n°77) or Qub 11a (samples n°106 and 30) did not show any amplification, were not included in the MIRU-VNTR analysis (S1 Table). This technique provided a Hunter-Gaston discriminatory Index (HGDI) of 0.977. The most discriminatory loci were ETR-A, ETR-B, QUB 11a, QUB 11b, QUB 26 and QUB 3232, while ETR-C and MIRU 4 loci provided a low index (HGDI <0.4 (Table 2)). The number of alleles ranged from one to six according to the loci (Table 2). In total, 20 clusters were shared by two or more isolates, including a total of 69/107 isolates (64.5%) giving a clustering rate (CR) of 45.8%. Each of the other 38 isolates (35.5%) had its a specific pattern. The largest clusters consisted of 11 samples (P13) and the smallest clusters comprised two isolates (P3, P42, P36, P33, P44, P51, P53, P58, and P11). The other ten clusters included seven (P60), six (P35), four (P7, P43, P9) and three (P32, P25, P23, P15, P47) isolates.

**Table 2:** Number of alleles and Hunter-Gaston discriminatory Index (HGDI) for the 8 MIRU-VNTR loci used in the study.

Loci	N° of alleles	HGDI
	(N° of isolates = 107)	(N° of isolates = 107)
ETR-A*	7	0.70
ETR-B	6	0.71
ETR-C	3	0.27
Qub 11a**	6	0.62
Qub 11b	5	0.62
Qub 3232	6	0.56
Qub 26	5	0.64
MIRU 4	3	0.15
HGDI including all the MIRU-VNTR data		0.977

*: excluding one strain without ETR-A allele

**: excluding one strain without Qub11a allele

Concerning the four isolates taken from Libyan patients, three were not clustered (P52, P54 and P21) and one shared the P60.

The P3 MIRU-VNTR profile included two *M*. *caprae*. The two other *M*. *caprae* had specific pattern**s** (P4 and P10).

The genetic analyses were performed on 102 *M*. *bovis* isolates using MIRU-VNTR data (excluding *M*. *caprae* isolates and isolates from Libyan patients). The genotypic diversity (*Gd*) and the mean genetic diversity (*H*_*S*_) were 0.53 and 0.52, respectively.

Globally, the genetic differentiations estimated by *F*st calculations were very low for the governorates and regions with a global genetic differentiation of 0.025 and 0.042, respectively. No genetic differentiation was found between *M*. *bovis* populations of the different governorates (Table 3). The only significant differentiation was observed between the South East (Tataouine, Medenine) and Central West (Gafsa, Sidi Bouzid, and Kasserine) regions of Tunisia (Table 4).

**Table 3:** Genetic differentiation (*F*_*st*_ index) between *M*. *bovis* populations of the different governorates.

	Gabes	Tataouine	Medenine	Gafsa	Sidi Bouzid	Kasserine
Sfax	0.026	0.037	0.006	0.011	ND	ND
Gabes	ND	0.076	0.016	0.058	0.066	0.047
Tataouine		ND	ND	0.016	0.034	0.020
Medenine			ND	0.026	0.044	0.015
Gafsa				ND	ND	ND
Sidi Bouzid					ND	ND
Kasserine						ND

ND: No Differentiation, *F*st was ≤ 0. No *Fst* value was significant

**Table 4:** Genetic differentiation (*F*_*st*_ index) between *M*. *bovis* populations of the different regions.

	Central West region (Gafsa, Sidi bouzid, Kasserine)	South East region (Tataouine, Medenine)
Central East region (Sfax, Gabes)	0.041	0.035
Central West region (Gafsa, Sidi bouzid, Kasserine)		0.057* (**P<0.05**)

*: *P* was statistically significant

### Combined analysis of MIRU-VNTR typing and spoligotyping

**Fig 2:**
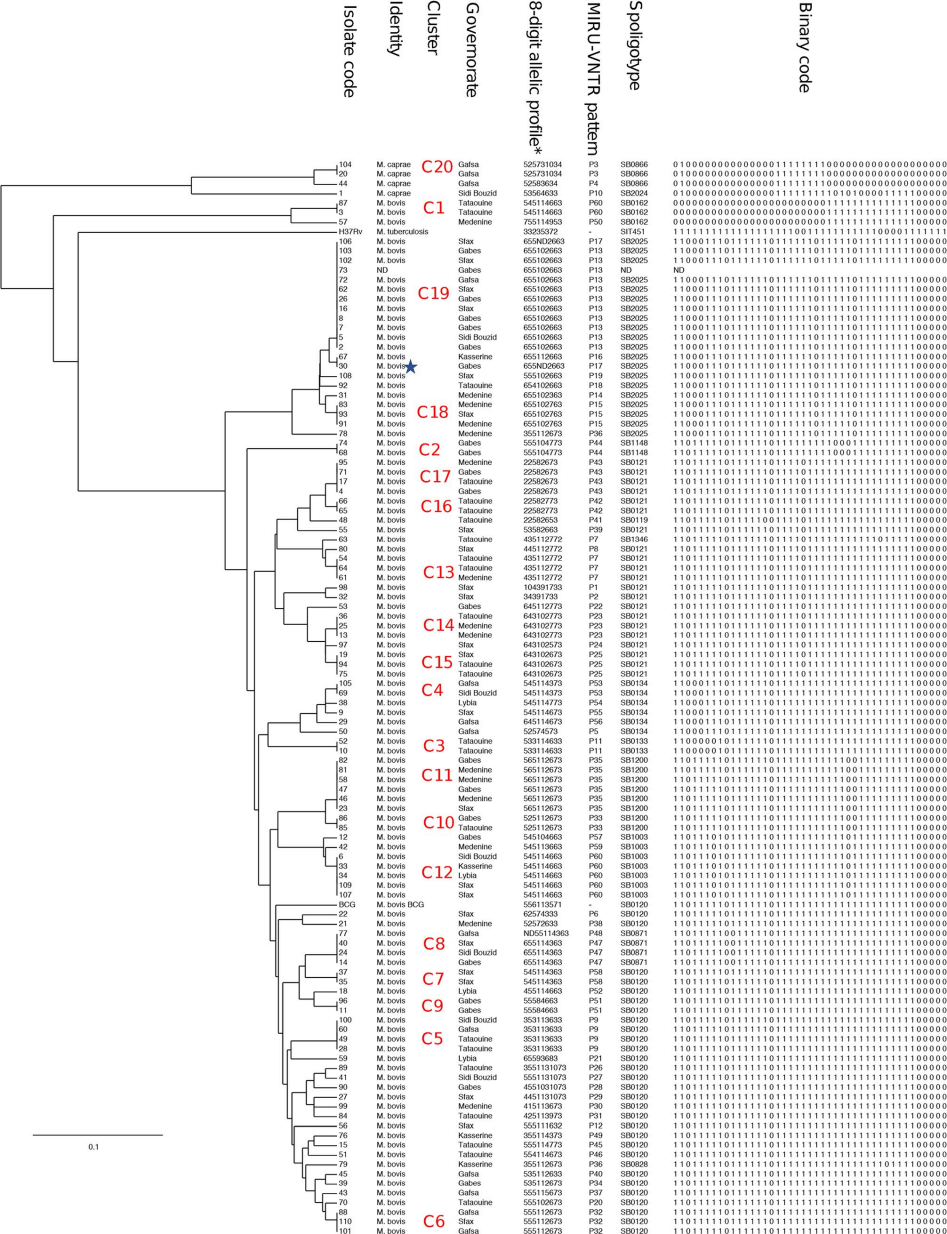
Dendogram built using Cavalli-Sforza and Edwards distance matrix calculated from the combination of MIRU-VNTR and spoligotyping data. The 20 clusters are identified on the tree as well as the species, the locality of isolates and the MIRU-VNTR and spoligotyping profiles. (Blue star) Sample n° 30 did not show any amplification for QUb11a, * 8-digit allelic profile corresponding to ETR-A, ETR-B, ETR-C, QUB 11a, QUB 11b, QUB 3232, QUB 26, and MIRU4.

Using the combined analysis of Spoligotyping and MIRU-VNTR methods, the 106 isolates revealed 61 different genotypes with a high level of discrimination (HGDI = 0.980). A total of 65 isolates (61.3%) were grouped in 20 clusters and the other 41 isolates (38.7%) had a unique pattern giving a CR of 42.45% (Fig 2, S1 Table). The cluster size ranged from 2 to 10 isolates per cluster. Out of these 20 clusters, four (C5, C6, C7, C9 with n = 4, 3, 2, 2, respectively) belonged to *M*. *bovis* SB0120 (BCG like) isolates. SB0121 was found in 5 clusters (C13, C14, C15, C16, C17 with n = 3, 3, 3, 2, 4, respectively). Two clusters (C18, C19) including SB2025 spoligotype were made up of 3 and 10 isolates, respectively. Two clusters (C10, C11) of SB1200 spoligotype included 2 and 6 isolates, respectively.

Two other clusters (C8, C12) were characterized by the spoligotypes SB0871 and SB1003 including three and five isolates, respectively. For the isolates from Libyan patients, three were not clustered and one belonged to SB1003 which was clustered (C12) with four Tunisian isolates (two from Sfax, one from Sidi Bouzid and one from Kasserine).

SB0134 was represented only in one cluster (C4) including two isolates. Three clusters (C1, C2, C3) included two isolates with the spoligotypes SB1062, SB1148, and SB0133, respectively.

Only one cluster (C20) including two isolates belonged to *M*. *caprae* (SB0866). The two other *M*. *caprae* (SB0866, SB2024) were not clustered.

### Comparison of Tunisian patients data with Tunisian cattle data

In Tunisia, 13/15 (86.7%) spoligotypes detected in our study were reported in cattle samples collected from November 2014 to April 2015 by Djemal *et al* [9]. From these 13 spoligotypes, seven (SB0120, SB0121, SB2025, SB1200, SB1003, SB0134 and SB2024) were previously identified by Lamine-khmiri *et al* in animal samples collected between 1991–2012 [10] and two (SB1003 and SB1200) in milk samples [28].

The six most detected *M*. *bovis* spoligotypes in our human isolates were also reported in cattle in Tunisia [9, 10] with different frequencies (S2 Table).

The SB0120 spoligotype found in 29 out of 109 (26.6%) Tunisian human isolates, was detected with a higher frequency in the cattle in the south of Tunisia as demonstrated by Djemal *et al* (36.4%) and Lamine-khmiri *et al* (37.1%) [9, 10] (S2 Table).

SB0121, SB2025, SB1200 were more prevalent in our study (21/110 (19.3%), 20/110 (19%) and 8/110 (7.3%), respectively) compared to the studies of Djemal *et al* [9] and Lamine-khmiri *et al* [10] (S2 Table). SB1200 was detected in cattle but also in one milk sample as previously reported in Tunisia [9, 10, 28]. SB1003 was detected in milk samples and in the cattle in Tunisia [9, 10, 28]. SB0134 identified as the second predominant spoligotype in cattle in Tunisia (11.4% [9] and 20% [10]), was detected only in 5.5% of our human isolates (S2 Table). Regarding *M*. *caprae* isolates, SB2024 was reported in human and cattle Tunisian isolates [9, 10] but SB0866 was not found in cattle in Tunisia (S2 Table).

It is worth noting that the spoligotypes observed in our isolates and shared with cattle (S2 Table), were mainly associated with raw milk-consuming patients and in contact with livestock. Indeed, 23/29 (79.3%) and 14/21 (66.7%) of the SB0120 and SB0121, respectively, were taken from patients who consumed raw milk and/or had contact with livestock. SB2025 was detected in 13/20 (65%) of raw milk-consuming patients of which one was in close contact with livestock. SB1200 and SB0134, were also detected in raw milk-consumers (5/8 (62.5%), 3/6 (50%), respectively) or in close contact to livestock (3/8 (37.5%), 3/6 (50%), respectively). For *M*. *caprae* isolates, SB0866 were detected in raw milk-consuming patients (3/3 (100%)) of whom 2 were in close contact with goats. SB2024 was found in patient in close contact with livestock (1/1 (100%)).

The comparison of the MIRU-VNTR genotypes based on the 6 common loci (ETR-A, ETR-B, QUB 11a, QUB 11b, QUB 3232 and MIR U4 (ETRD) between human and cattle isolates from Tunisia showed a total of 14 common VNTR profiles (CVP) (Table 5, S2 Table). Among them, 13 CVP were recently identified in cattle from Tunisia by Djemal *et al* [9]. CVP 1, 2, 10, 11, 12 and 14 were also previously detected in Tunisian cattle isolates [10] (Table 5 and S2 Table). It is worth noting that 13/14 (92.85%) and 9/14 (64.3%) CVP were detected in our isolates from patients who consumed raw milk (35/73 (47.9%)) or who were in close contact with livestock (12/34 (35.3%)), respectively.

**Table 5:** Comparison of the distribution of common VNTR profiles between our study and previous studies in cattle in Tunisia [9, 10].

Common VNTR Profile	Genotype	This study	Lamine-Khemiri *et al*. 2013 [10]	Djemal *et al* 2017 [9]
CVP1	5.4.11.4.6.3	+	+	+
CVP2	5.6.11.2.6.3	+	+	+
CVP3	3.5.11.2.6.3	+		+
CVP4	5.5.11.4.6.3	+		+
CVP5	6.4.10.2.7.3	+		+
CVP6	5.5.11.5.6.3	+		+
CVP7	4.3.11.2.7.2	+		+
CVP8	4.5.11.4.6.3	+		+
CVP9	5.4.11.4.3.3	+		+
CVP10	5.5.11.2.6.3	+	+	+
CVP11	5.3.11.2.6.3	+	+	+
CVP12	3.5.11.3.6.3	+	+	+
CVP13	6.5.10.2.6.3	+		+
CVP14	6.4.11.2.7.3	+	+	

CVP: Common VNTR profile based on the following VNTR loci (ETR-A, ETR-B, QUB 11a, QUB 11b, QUB 3232 and MIRU 4) of strains from our study and previously published Tunisian data by Lamine-Khemiri *et al* [10] and Djemal *et al* [9].

### Recent transmission rate and risk factor analysis

The recent transmission rate among the 98 *M*. *bovis* isolates from Tunisia (excluding *M*. *caprae* isolates (n = 4), isolates from Libyan patients (n = 4) and isolates with missing genotyping data (n = 4)) was 43.9% based on the MIRU-VNTR patterns plus spoligotyping. The unique genotypes were detected in children (0–4 years), in old patients (≥ 60 years), in patients from Sidi bouzid, Kasserine and in patients with personal TB history and with no BCG vaccination (S3 Table).

The clustering was then analyzed in function of the demographic and clinical data (S3 Table). The number of isolates in clusters was high and statistically significant in female patients (75.8%), in patients between 15 to 59 years (79%), in patients with urban lifestyle (72.6%), in patients living in Gabes (24.2%), Sidi Bouzid (8.1%) and in Central Eastern Tunisia (43.5%) (S3/A Table). The univariate statistical analysis showed that the clustering was not affected by age, sex, area of residence, raw and/or unpasteurized milk consumption, contact with livestock, personal TB history and BCG vaccination status (*P* >0.05) (S3 Table).

Considering the three main *M*. *bovis*, SB0120, SB0121 and SB2025 (corresponding to 66 patients) detected in our samples, the *G*d (0.58), the *H*_S_ (0.12) and the CR (42.4%) values were equivalent to the data obtained in the whole Tunisian *M*. *bovis* samples based on the MIRU-VNTR patterns plus spoligotyping (S4 Table). Among the 66 patients infected by the three main *M*. *bovis* SBs (SB0120, SB0121 and SB2025), the univariate analysis showed that the clustering was statistically associated with female patients (87.2% vs 63%, *p* = 0.021) but not affected by age, area of residence, raw and/or unpasteurized milk consumption, contact with livestock, personal TB history and BCG vaccination status (*p* >0.05) (S4 Table).

## Discussion

In South Tunisia, almost 77% of clinically and bacteriologically diagnosed cases of EPTB are attributed to *M*. *bovis* [7]. To our knowledge, this is the first study that investigates molecular characterization of *M*. *bovis* causing human EPTB in the South of Tunisia.

The geographical distribution of human EPTB cases due to *M*. *bovis* in our study showed that the disease was widespread in the seven governorates in Southern Tunisia.

SB0120, SB0121 and SB0134 have been described in humans and livestock across the globe [29; 30] indicating the zoonotic importance of these *M*. *bovis* spoligotypes. The most detected spoligotype in our study was SB0120 (26.9%) which is also the most common in humans in Italy (63.8%) [31], France (69%) [32], Germany (20%) [33] compared to 1% in the United Kingdom [34]. SB0120 is also detected in cattle in some European countries like France [35], Italy [30], Portugal [30, 36], Spain [37], and Germany [33] as well as in neighboring countries (Algeria and Morocco) [38, 39].

The frequency of SB0121 (19.3%) among our human isolates was in agreement with those mentioned in humans in previous studies (e.g. France (15%) [32] and Spain (14.1%) [40]). However, it was scarce in human *M*. *bovis* isolates from individuals in London and the southeast of England (6%) [29]. SB0134, detected in our study at 5.5%, was found in human *M*. *bovis* cases in France [32], in Spain (10.5% to 13.1%) [40; 41], in London, in the southeast of England (6%) [29], in UK (2%) [34] and in Italy (2%) [31]. SB0121 and SB0134 were previously detected in animal isolates all over the world [30] including neighboring countries such as Algeria [38] and Morocco [39]. Interestingly, this is the first study to identify SB2025 and SB1200 in human *M*. *bovis* isolates causing EPTB. It is worth noting that these two spoligotypes were isolated for the first time in milk and cattle samples in Tunisia, respectively [10; 28] and they were not detected in cattle in France [35].

Over the previous years, more emphasis was put on human infection due to *M*. *caprae* because of the increasing implication of this species in TB infection in cattle or other animals [42; 43]. Among our isolates, *M*. *bovis* subsp. *caprae* SB2024 was represented by one isolate and was previously found in Tunisian isolates from cattle [9, 10]. This spoligotype was never reported in humans. In our study, *M*. *bovis* subsp. *caprae* SB0866 was localized in the same region (Gafsa in Central West Tunisia) but was never found in Tunisian cattle samples [9, 10]. Only one EPTB case infected by this spoligotype was reported in humans in Germany [33].

The proportion of spoligotypes detected in our human isolates and shared with those previously found in cattle in Tunisia was of 86.7%. This was in agreement with the high values (61–90.9%) previously published [44, 45]. Nevertheless, there are differences in frequency of certain spoligotypes among human and cattle isolates reported in Tunisia. For example, SB0134 was the second predominant spoligotype (11.4% to 20%) [9, 10] in cattle while it was the fifth most common (5.5%) in our human strains. This finding suggests differential abilities of spoligotypes to infect humans and animals [30, 44, 45]. Regarding MIRU-VNTR data, human isolates shared some CVP genotypes based on the 6 common loci (ETR-A, ETR-B, QUB 11a, QUB 11b, QUB 3232 and MIR U4 (ETR-D) with cattle in Tunisia. However, humans and cattle showed different *M*. *bovis* populations suggesting a host-specific pool of genotypes as described in the Table 5. All these findings showed a specificity of *M*. *bovis* populations in terms of geography and hosts as observed for *M*. *tuberculosis*. It would be pertinent to go further in the evolutionary and biological mechanisms linked to host specificity in order to better understand the transmission mechanism between humans and cattle.

As previously reported in Tunisia [6, 7], our results underlined that human EPTB cases due to *M*. *bovis* were more common in women [6, 7, 46, 47]. Female gender represents a major risk factor for EPTB according to several published reports in Turkey, USA, Asia, Egypt and North Africa [48, 49; 50; 51]. The sociodemographic data in our current study showed that EPTB was also more frequent in “younger patients” between 15–59 years of whom 49% ranged between 20 and 40 years and 81% were female. This finding is in agreement with data reported in Tunisia as well as in several areas with moderate or high TB burden [6, 7, 46, 47, 49, 51, 52, 53, 54]. This can be explained by a higher consumption of raw milk or derivatives among “young people” than the elderly. Furthermore, due to the current lifestyle, females are generally more exposed to livestock or products from cattle. Indeed, our data showed that female patients are more than twice as likely as the male patients to have contact with livestock or other animals and 68% of “young patients” (ranging between 15–59 years) declared to consume raw milk or derivatives of whom almost 80% were females. Furthermore, the consumption of unpasteurized milk and dairy products has been indicated as the most likely source of transmission in clinical cases of LTB, which is the main form of zoonotic TB. Of note, most of our patients (73/110, (66.4%)) are consumers of raw milk or derivatives, whereas only 34/110 (30.9%) patients had contact with livestock, suggesting that, in our study, dairy products are one of the main sources of *M*. *bovis* human infection. This could explain the increased proportion of cases with positive LTB diagnosis (100/110 (91%)) caused by *M*. *bovis* among EPTB patients in Southern Tunisia. In these areas, people working in dairy farms commonly take raw milk home and sell it clandestinely to markets, increasing the risk of zoonotic TB transmission to humans. It has been shown that *M*. *bovis* was able to survive and to remain virulent for extended periods in a variety of cheeses made with raw milk [55; 56; 57].

MIRU-VNTR typing method showed a high genotypic and genetic diversity with 6/8 loci showing high allelic diversity and HGDI values. In our study, the global genetic differentiations based on MIRU-VNTR were very low between governorates and regions. Nevertheless, it was significant between populations from the South East (Tataouine, Medenine) and the Central West (Gafsa, Sidi Bouzid, Kasserine) regions of Tunisia. These findings suggest a slight structuring in the *M*. *bovis* population in humans. These findings are in concordance with those of Smith *et al* who mentioned that some *M*. *bovis* genotypes could be more localized compared to others [58]. The diversity observed in the South East (Tataouine, Medenine) and the Central West regions could be explained by the fact that these two regions are transit areas for livestock traffic through the Sahara coming from different neighboring areas with high bTB burden (e.g. Arab Maghreb (Libya and Algeria) and African countries).

Using combined spoligotyping and MIRU-VNTR method, a high clustering rate of 43.9% was obtained. These data do not corroborate previous reports showing much lower rates for LTB, 29.5% [59] and 35% [60] in Southwest Ethiopia and 33% for *M*. *bovis* TB infection in United Kingdom [61]. These results suggest a high level of recent transmission of zoonotic bTB in humans in Tunisia. SB0120, SB0121 and SB2025 represent the spoligotypes harboring the highest frequencies of isolates among clustered strains (42.4%). This indicates that the predominant spoligotypes, SB0120, SB0121 and SB2025, are mainly responsible for recent transmissions in Southern Tunisia.

According to the clustering analysis, female patients, patients aged between 15–59 years, from Central East Tunisia (Sfax, Gabes), South East regions, patients who not have a personal TB history and who have a positive BCG vaccination status were significantly clustered cases. Up to 60% and 58% of isolates from EPTB patients who consumed raw milk/milk products and who were in close contact to livestock, respectively, were also highly regrouped in clusters. Among human isolates that shared CVP genotypes with cattle isolates, 75.5% were in clusters. To explain these recent human transmission cases, oral transmission route seems evident since 72/73 (98.6%) and 33/34 (97%) patients who consumed raw milk/derivatives or who had animal contact, respectively, had extrapulmonary manifestations in Lymph nodes.

It is worth noting that, from the analysis, the elderly, children, young population, patients with personal TB history and with no BCG vaccination were significantly more associated with non-clustered isolates, which can be interpreted as ancient infection. Nevertheless, the recent transmission rate can be underestimated since only an exhaustive study including human and animal isolates could rigorously determine the bTB epidemiology.

Furthermore, our study was limited to the South of Tunisia, and the majority of patients were from urban areas. Thus, a larger study with sampling combining rural and urban areas from both North and South of the country, humans and animals would provide a more accurate view of *M*. *bovis* molecular epidemiology. This kind of study would give a better evaluation of bTB transmission risk to humans and a better identification of the sources and routes of transmission of *M*. *bovis* in humans in Tunisia. Besides, the complementary detailed genomic analysis would also allow study the genes/proteins involved in the biological processes responsible for host specificity and/or pathogenesis of the isolates.

In conclusion, our study described for the first time, in southern Tunisia, the transmission of zoonotic TB to humans due to *M*. *bovis*. Our findings underlined that recent transmission was the possible explanation of most *M*. *bovis* EPTB infections. The high genetic diversity reflects a large number of contamination sources. Nevertheless, the high clustering rates of certain genotypes such as the SB0120 and the strong association with milk or milk products consumption suggest that some genotypes have a higher ability to infect humans and that the source is mainly linked to oral ingestion.

Our study highlighted the urgent need for a better veterinary control with the implementation of effective and comprehensive strategies in order to reach an effective protection not only of humans but also of animals.

## Supporting information

S1 Table Excel spreadsheet with raw data. (XLS)

S2 Table Comparison of *M. bovis* isolates data from Tunisian patients with those from Tunisian cattle. (DOC)

S3 Table Genetic data in function of the demographic and geographic characteristics (A) risk factors (B) of patients with extrapulmonary tuberculosis from Tunisia and with the 3 major detected SB (C).
(DOC)

S4 Table Genetic data in function of the demographic and geographic characteristics (A) and risk factors of EPTB patients caused by Mycobacterium bovis bearing major SB (SB0120, SB0121 and SB2025).
(DOC)
