# Supplementary material for: A first insight into genetic diversity of Mycobacterium bovis isolated from extrapulmonary tuberculosis patients in South Tunisia assessed by spoligotyping and MIRU VNTR
Source: PLoS Negl Trop Dis. 2019 Sep 18;13(9):e0007707. doi: 10.1371/journal.pntd.0007707 (PMC6750577; doi:10.1371/journal.pntd.0007707)
Supplement: S2 Table — (DOC) [file pntd.0007707.s002.doc]

**S2 Table: Comparison of *M. bovis* isolates data from Tunisian patients with those from Tunisian cattle**

| *Study* | Source, (Nb*) | Nb isolates€ | Region/  Nb | | Total of genotypes | Common VNTR loci/Common Genotypes, | | | | | | VNTR  types | HGDI | Total of cluster | Total of spoligotypes | Common frequent spoligotypes | | | | | | | |
| --- | --- | --- | --- | --- | --- | --- | --- | --- | --- | --- | --- | --- | --- | --- | --- | --- | --- | --- | --- | --- | --- | --- | --- |
|  |  |  |  | |  | ETR-A | ETR-B | QUB11a | QUB11b | QUB3232 | MIRU4 |  |  |  |  | SB0120  Nb (%)  Region  (Nb) | SB 0121  Nb (%)  Region  (Nb) | SB2025  Nb (%)  Region (Nb) | SB1200  Nb (%)  Region  (Nb) | SB1003  Nb (%)  Region  (Nb) | SB0134  Nb (%)  Region  (Nb) | SB2024  Nb (%)  Region  (Nb) | SB0866  Nb (%)  Region  (Nb) |
| *This study* | Human  (110) | 110 | **Sf, G**, Ta, Me, Gf, Ke SB :7 and Ly | | 60 | 5 | 4 | 11 | 4 | 6 | 3 | CVP1 | 0,977 | 20 | 15/109 | 29  (26.6%)  Ta(7)  Sf(6)  Gf(5)  G(4)  SB(2)  Me(2)  Ke(1)  Ly(2) | 21  (19.3%)  Ta(8)  Sf(5)  Me(4)  G(3) | 20  (18.3%)  Sf(6)  G(6)  Me(4)  Gf(1)  Ke(1)  SB(1)  Ta(1) | 8  (7.3%)  Sf(1)  G(3)  Me(3)  Ta(1) | 7  (6.4%)  Sf(2)  G(1)  SB(1)  Me(1)  Ke(1)  Ly (1) | 6  (5.5%)  Gf(3)  Sf(1)  SB(1)  Ly(1) | 1  (0.9%)  SB(1) | 3  (2.7%)  Gf(3) |
|  |  |  |  |  |  | 5 | 6 | 11 | 2 | 6 | 3 | CVP2 |  |  |  |  |  |  |  |  |  |  |  |
|  |  |  |  |  |  | 3 | 5 | 11 | 2 | 6 | 3 | CVP3 |  |  |  |  |  |  |  |  |  |  |  |
|  |  |  |  |  |  | 5 | 5 | 11 | 4 | 6 | 3 | CVP4 |  |  |  |  |  |  |  |  |  |  |  |
|  |  |  |  |  |  | 6 | 4 | 10 | 2 | 7 | 3 | CVP5 |  |  |  |  |  |  |  |  |  |  |  |
|  |  |  |  |  |  | 5 | 5 | 11 | 5 | 6 | 3 | CVP6 |  |  |  |  |  |  |  |  |  |  |  |
|  |  |  |  |  |  | 4 | 3 | 11 | 2 | 7 | 2 | CVP7 |  |  |  |  |  |  |  |  |  |  |  |
|  |  |  |  |  |  | 4 | 5 | 11 | 4 | 6 | 3 | CVP8 |  |  |  |  |  |  |  |  |  |  |  |
|  |  |  |  |  |  | 5 | 4 | 11 | 4 | 3 | 3 | CVP9 |  |  |  |  |  |  |  |  |  |  |  |
|  |  |  |  |  |  | 5 | 5 | 11 | 2 | 6 | 3 | CVP10 |  |  |  |  |  |  |  |  |  |  |  |
|  |  |  |  |  |  | 5 | 3 | 11 | 2 | 6 | 3 | CVP11 |  |  |  |  |  |  |  |  |  |  |  |
|  |  |  |  |  |  | 3 | 5 | 11 | 3 | 6 | 3 | CVP12 |  |  |  |  |  |  |  |  |  |  |  |
|  |  |  |  |  |  | 6 | 5 | 10 | 2 | 6 | 3 | CVP13 |  |  |  |  |  |  |  |  |  |  |  |
|  |  |  |  |  |  | 6 | 4 | 11 | 2 | 7 | 3 | CVP14 |  |  |  |  |  |  |  |  |  |  |  |
|  |  |  |  | |  |  |  |  |  |  |  |  |  |  |  |  |  |  |  |  |  |  |  |
| *Djemal et al [9]* | Cattle  (149) | 96 | **Sf, G,**  **SB,** k, B, Je, Ma, Mo, A, M:10 | | 73 | 5 | 4 | 11 | 4 | 6 | 3 | CVP1 | 0,993 | 15 | 25/96 | 35 (36.4%)  Sf(24)  M(4) Ma(2)  Sb(3)  Mo(1)  k(1) | 7  (7.2%)  Sf(4)  G(1)  M(1)  SB(1) | 7  (7.2%)  Sf(4)  G(1)  Ma(1)  M(1) | 4  (4.1%)  Sf(2)  Je(1)  B(1) | 6  (6.2%)  Sf(5)  Ma(1) | 11  (11.4%)  Sf(7)  M(3)  A(1) | 1  (1%)  Sf(1) | NF |
|  |  |  |  |  |  | 5 | 6 | 11 | 2 | 6 | 3 | CVP2 |  |  |  |  |  |  |  |  |  |  |  |
|  |  |  |  |  |  | 3 | 5 | 11 | 2 | 6 | 3 | CVP3 |  |  |  |  |  |  |  |  |  |  |  |
|  |  |  |  |  |  | 5 | 5 | 11 | 4 | 6 | 3 | CVP4 |  |  |  |  |  |  |  |  |  |  |  |
|  |  |  |  |  |  | 6 | 4 | 10 | 2 | 7 | 3 | CVP5 |  |  |  |  |  |  |  |  |  |  |  |
|  |  |  |  |  |  | 5 | 5 | 11 | 5 | 6 | 3 | CVP6 |  |  |  |  |  |  |  |  |  |  |  |
|  |  |  |  |  |  | 4 | 3 | 11 | 2 | 7 | 2 | CVP7 |  |  |  |  |  |  |  |  |  |  |  |
|  |  |  |  |  |  | 4 | 5 | 11 | 4 | 6 | 3 | CVP8 |  |  |  |  |  |  |  |  |  |  |  |
|  |  |  |  |  |  | 5 | 4 | 11 | 4 | 3 | 3 | CVP9 |  |  |  |  |  |  |  |  |  |  |  |
|  |  |  |  |  |  | 5 | 5 | 11 | 2 | 6 | 3 | CVP10 |  |  |  |  |  |  |  |  |  |  |  |
|  |  |  |  |  |  | 5 | 3 | 11 | 2 | 6 | 3 | CVP11 |  |  |  |  |  |  |  |  |  |  |  |
|  |  |  |  |  |  | 3 | 5 | 11 | 3 | 6 | 3 | CVP12 |  |  |  |  |  |  |  |  |  |  |  |
|  |  |  |  |  |  | 6 | 5 | 10 | 2 | 6 | 3 | CVP13 |  |  |  |  |  |  |  |  |  |  |  |
|  |  |  |  | |  |  |  |  |  |  |  |  |  |  |  |  |  |  |  |  |  |  |  |
| *L-K et aL [10]* | Cattle, (100) | 35 | **Sf, G**, B, **SB**, N :5 | | 19 | 5 | 4 | 11 | 4 | 6 | 3 | CVP1 | 0.969 | 6 | 9/35 | 16  (45.7%)  Sf(11)  B(2)  SB(2)  G(1) | 1  (2.8%)  Sf(1) | 3  (8.6%)  Sf(3) | 1  (2.8%)  Sf(1) | 2 (5.7%)  Sf(2) | 7  (20%)  Sf(7) | 1  (2.8%)  N(1) | NF |
|  |  |  |  |  |  | 5 | 6 | 11 | 2 | 6 | 3 | CVP2 |  |  |  |  |  |  |  |  |  |  |  |
|  |  |  |  |  |  | 5 | 5 | 11 | 2 | 6 | 3 | CVP10 |  |  |  |  |  |  |  |  |  |  |  |
|  |  |  |  |  |  | 5 | 3 | 11 | 2 | 6 | 3 | CVP11 |  |  |  |  |  |  |  |  |  |  |  |
|  |  |  |  |  |  | 3 | 5 | 11 | 3 | 6 | 3 | CVP12 |  |  |  |  |  |  |  |  |  |  |  |
|  |  |  |  |  |  | 6 | 4 | 11 | 2 | 7 | 3 | CVP14 |  |  |  |  |  |  |  |  |  |  |  |
|  |  |  | |  | | | | | | | | | | | | | | | | | | | |

*Nb : Number ;* €*: M.bovis isolates****;*** *Sf : Sfax ; G : gabes ; Ta : Tataouine, Me : Medenine, Gf : gafsa ; SB : Sidi Bouzid ; Ke : Kasserine ; M : Mahdia, K : kairaouen, B : Beja, Je : Jendouba, Ma : Manouba, Mo : Monastir, T : Tunis, N : Nabeul, A : Ariana, Ly: Lybia. Common region between the three Tunisian studies are in fold. CVP: Common VNTR profile.*
