# Supplementary material for: A first insight into genetic diversity of Mycobacterium bovis isolated from extrapulmonary tuberculosis patients in South Tunisia assessed by spoligotyping and MIRU VNTR
Source: PLoS Negl Trop Dis. 2019 Sep 18;13(9):e0007707. doi: 10.1371/journal.pntd.0007707 (PMC6750577; doi:10.1371/journal.pntd.0007707)
Supplement: S3 Table — Genetic data in function of the demographic and geographic characteristics (A) risk factors (B) of patients with extrapulmonary tuberculosis from Tunisia and with the 3 major detected SB (C). (DOC) [file pntd.0007707.s003.doc]

**Table S3. Genetic data in function of the demographic and geographic characteristics (A) risk factors (B) of patients with extrapulmonary tuberculosis from Tunisia and with the 3 major detected SB (C)**

**(A) Demographic and geographic characteristics**

| **Patients characteristics** | **N°**  **of isolates**  **(%)** | **Genotypic diversity (Gd^a^)*** | **Mean**  **genetic**  **diversity (Hs)*** | **N°of clusters*** | **N°**  **of clustered isolates (%) *** | **N°**  **of Non-clustered isolates (%)** | **Recent Transmission rate**  **(%)*** | **N°**  **of SB0120** | **N°**  **of SB0121** | **N°**  **of SB2025** | **N°**  **of 1200** | **N°**  **of SB1003** | **N°**  **of SB0134** |
| --- | --- | --- | --- | --- | --- | --- | --- | --- | --- | --- | --- | --- | --- |
| **Total patients** | 110  (100%) | 52/98*(0.53) | 0.15 | 19 | 62  (63.3) | 36  (36.7) | 43.9 | 27 | 21 | 18 | 8 | 6 | 5 |
| **Sex** |  |  |  |  |  |  |  |  |  |  |  |  |  |
| Males | 33  (30%) | 20/29 (0.7) | 0.16 | 9 | 15/62  (24.2) | 14/36  (38.9) | 20.7 | 8 | 3 | 4 | 5 | 2 | 3 |
| Females | 77  (70%) | 38/69 (0.55) | 0.15 | 17 | 47/62  (75.8) | 22/36  (61.1) | 43.5 | 19 | 18 | 14 | 3 | 4 | 2 |
| **Age** |  |  |  |  |  |  |  |  |  |  |  |  |  |
| 0-4 years | 8 (7.3%) | 8/8  (1) | 0.12 | 4 | 4/62  (6.5) | 4/36  (11.1) | 0 | 3 | - | 2 | 1 | - | - |
| 5-14 years | 13 (11.8%) | 12/13  (0.92) | 0.18 | 7 | 7/62  (11.3) | 6/36  (16.7) | 0 | 3 | 1 | 2 | 2 | 2 | 1 |
| 15-59 years | 84 (76.4%) | 42/74  (0.56) | 0.15 | 19 | 49/62  (79) | 25/36  (69.4) | 40.5 | 20 | 19 | 14 | 5 | 4 | 4 |
| ≥ 60 years | 5 (4.5%) | 3/3 (1) | 0.11 | 3 | 2/62  (3.2) | 1/36  (2.8) | 0 | 1 | 1 | - | - | - | - |
| **Life style** |  |  |  |  |  |  |  |  |  |  |  |  |  |
| urban | 79 (71.8%) | 44/73 (0.6) | 0.16 | 19 | 45/62  (72.6) | 28/36  (77.8) | 35.6 | 20 | 17 | 16 | 5 | 3 | 2 |
| rural | 31 (28.2%) | 20/25  (0.8) | 0.12 | 13 | 17/62  (27.4) | 8/36  (22.2) | 16 | 7 | 4 | 2 | 3 | 3 | 3 |
| **Origin** |  |  |  |  |  |  |  |  |  |  |  |  |  |
| Central East Tunisia (Sfax, Gabes) | 44 (40%) | 26/41 (0.63) | 0.13 | 11 | 27/62  (43.5) | 14/36  (38.9) | 39 | 10 | 9 | 10 | 4 | 3 | 1 |
| Central west Tunisia (Gafsa, Sidi bouzid, Kasserine) | 24 (21.8%) | 14/19 (0.74) | 0.11 | 7 | 11/62  (17.7) | 8/36  (22.2) | 21.1 | 8 | - | 3 | - | 2 | 4 |
| South east Tunisia (Tataouine, Medenine,) | 38 (34.5%) | 24/38  (0.63) | 0.18 | 11 | 24/62  (38.7) | 14/36  (38.9) | 34.2 | 9 | 12 | 5 | 4 | 1 | - |
| Sfax | 23 (21%) | 17/22 (0.77) | 0.14 | 8 | 12/62  (19.4) | 10/36  (27.8) | 18.2 | 6 | 6 | 5 | 1 | 2 | 1 |
| Gabes | 21 (19%) | 10/19 (0.53) | 0.13 | 7 | 15/62  (24.2) | 4/36  (11.1) | 42.1 | 4 | 3 | 5 | 3 | 1 | - |
| Tataouine | 23 (21%) | 16/23 (0.69) | 0.19 | 9 | 15/62  (24.2) | 8/36  (22.2) | 26.1 | 7 | 8 | 1 | 1 | - | - |
| Medenine | 15 (13.6%) | 11/15  (0.73) | 0.18 | 5 | 9/62  (14.5) | 6/36  (16.7) | 26.7 | 2 | 4 | 4 | 3 | 1 | - |
| Gafsa | 13  (11.8%) | 8/9 (0.88) | 0.10 | 6 | 5/62  (8.1) | 4/36  (11.1) | 0 | 5 | - | 1 | - | - | 3 |
| Sidi bouzid | 7 (6.4%) | 6/6 (1) | 0.13 | 5 | 5/62  (8.1) | 1/36  (2.8) | 0 | 2 | - | 1 | - | 1 | 1 |
| Kasserine | 4 (3.6%) | 4/4 (1) | 0.13 | 1 | 1/62  (1.6) | 3/36  (8.3) | 0 | 1 | - | 1 | - | 1 | - |

**(B) Risk factors**

| **Risk factors** | **N°**  **of isolates (%)** | **Genotypic d**  **iversity (Gd^b^)** | **Mean**  **genetic**  **diversity (Hs)** | **N°**  **of clusters** | **N°**  **of clustered isolates (%) *** | **N°**  **of Non-clustered isolates (%)** | **Recent Transmission rate**  **(%)*** | **N°**  **of SB0120** | **N°**  **of SB0121** | **N°**  **of SB2025** | **N°**  **of 1200** | **N°**  **of SB1003** | **N°**  **of SB0134** |
| --- | --- | --- | --- | --- | --- | --- | --- | --- | --- | --- | --- | --- | --- |
| **Raw milk consumption** |  |  |  |  |  |  |  |  |  |  |  |  |  |
| yes | 73 (66.4) | 40/67 (0.6) | 0.164 | 19 | 41/62  (66.1) | 26/36  (72.2) | 32.8 | 20 | 12 | 12 | 5 | 4 | 3 |
| No | 30 (27.3) | 20/25  (0.80) | 0.132 | 11 | 17/62  (27.4) | 8/36  (22.2) | 24 | 6 | 7 | 6 | 1 | 2 | 2 |
| No data | 7  (6.4) | 5/6  (0.83) | 0.11 | 3 | 4/62  (6.5) | 2/36  (5.6) | 16.7 | 1 | 2 | 0 | 2 | 0 | 0 |
| **Contact to livestock** |  |  |  |  |  |  |  |  |  |  |  |  |  |
| yes | 34 (30.9) | 22/26  (0.85) | 0.123 | 12 | 15/62  (24.2) | 11/36  (30.6) | 11.5 | 8 | 3 | 1 | 3 | 3 | 2 |
| No | 53 (48.2) | 30/51  (0.59) | 0.17 | 16 | 36/62  (58.1) | 15/36  (41.7) | 39.2 | 13 | 11 | 16 | 2 | 3 | 2 |
| No data | 23 (20.9) | 17/21  (0.81) | 0.13 | 8 | 11/62  (17.7) | 10/36  (27.8) | 14.3 | 6 | 7 | 1 | 3 | 0 | 1 |
| **TB history** |  |  |  |  |  |  |  |  |  |  |  |  |  |
| yes | 6  (5.4) | 5/5  (1) | 0.15 | 5 | 4/62  (6.5) | 1/36  (2.8) ^δ^ | 0 | 0 | 3 | 0 | 0 | 1 | 0 |
| No | 60 (54.5) | 39/56  (0.70) | 0.14 | 14 | 30/62  (48.4) | 26/36  (72.2) | 28.6 | 19 | 10 | 11 | 3 | 5 | 2 |
| No data | 44  (40) | 22/37  (0.59) | 0.167 | 18 | 28/62  (45.2) | 9/36  (25) | 27 | 8 | 8 | 7 | 5 | 0 | 3 |
| **BCG vaccination** |  |  |  |  |  |  |  |  |  |  |  |  |  |
| yes | 62 (56.4) | 35/57  (0.61) | 0.135 | 13 | 34/62  (54.8) | 23/36  (63.9) | 36.8 | 15 | 11 | 12 | 5 | 6 | 2 |
| No | 3  (2.7) | 3/3  (1) | 0.12 | 2 | 2/62  (3.2) | 1/36  (2.8) | 0 | 1 | 1 | 0 | 0 | 0 | 0 |
| No data | 45 (40.9) | 25/38  (0.66) | 0.18 | 15 | 26/62  (41.9) | 12/36  (33.3) | 28.9 | 11 | 9 | 6 | 3 | 0 | 3 |

**(C) Isolates from patients with each major detected SB**

| **SB** | **Genotypic diversity (Gd^a^)*** | **Mean**  **genetic**  **diversity (Hs)*** | **N°of clusters*** | **N°**  **of clustered isolates (%) *** | **N°**  **of Non-clustered isolates (%)** | **Recent Transmission rate**  **(%)*** | **N°**  **of SB0120** | **N°**  **of SB0121** | **N°**  **of SB2025** |
| --- | --- | --- | --- | --- | --- | --- | --- | --- | --- |
| **SB0120** | 20/27  (0.74) | 0.075 | 4 | 11/62  (17.74) | 16/36  (44.4) | 26 | 27 | - | - |
| **SB0121** | 11/21  (0.52) | 0.078 | 5 | 15/62  (24.2) | 6/36  (16.7) | 47.6 | - | 21 | - |
| **SB2025** | 7/18  (0.4) | 0.02 | 2 | 13/62  (21) | 5/36  (13.9) | 61.11% | - | - | 18 |
| **Other SB** | 16/32  (0.5) | 0.185 | 9 | 23/62  (37.1) | 9/36  (25) | 43.75% | - | - | - |

*****: Among the 110 isolates, 98 were used for the analyses done in Table S3 (*M. caprae* isolates, isolates from patients from Libya and strains with genotyping missing data (N°73, 77, 30, 106) were excluded).

^a^Gd : number of different genotypes / Total number of isolates in each population
