# Supplementary material for: A first insight into genetic diversity of Mycobacterium bovis isolated from extrapulmonary tuberculosis patients in South Tunisia assessed by spoligotyping and MIRU VNTR
Source: PLoS Negl Trop Dis. 2019 Sep 18;13(9):e0007707. doi: 10.1371/journal.pntd.0007707 (PMC6750577; doi:10.1371/journal.pntd.0007707)
Supplement: S4 Table — Genetic data in function of the demographic and geographic characteristics (A) and risk factors of EPTB patients caused by Mycobacterium bovis bearing major SB (SB0120, SB0121 and SB2025). (DOC) [file pntd.0007707.s004.doc]

**S4 Table. Genetic data in function of the demographic and geographic characteristics (A) and risk factors of EPTB patients caused by *Mycobacterium bovis* bearing major SB (SB0120, SB0121 and SB2025)**

**(A) Demographic and geographic characteristic**

| **Patients characteristics** | **N°**  **of isolates**  **(%)** | **Genotypic diversity (Gd^a^)*** | **Mean**  **genetic**  **diversity (Hs)*** | **N°of clusters*** | **N°**  **of clustered isolates (%) *** | **N°**  **of Non-clustered isolates (%)** | **Recent Transmission rate**  **(%)*** |
| --- | --- | --- | --- | --- | --- | --- | --- |
| Total patients | 110  (100%) | 38/66  (0.58) | 0.124 | 11 | 39 | 27 | 42.4 |
| Sex |  |  |  |  |  |  |  |
| Males | 33  (30%) | 13/15 (0.86) | 0.12 | 3 | 5/39  (12.8) | 10/27  (37) | 13.3 |
| Females | 77  (70%) | 28/51  (0.54) | 0.13 | 11 | 34/39  (87.2)^γ^ | 17/27  (63) ^γ^ | 45.1 |
| Age |  |  |  |  |  |  |  |
| 0-4 years | 8 (7.3%) | 5/5  (1) | 0.12 | 2 | 2/39  (5.1) | 3/27  (11.1) | 0 |
| 5-14 years | 13 (11.8%) | 6/6  (1) | 0.12 | 3 | 3/39  (7.7) | 3/27  (11.1) | 0 |
| 15-59 years | 84 (76.4%) | 31/53  (0.58) | 0.13 | 11 | 33/39  (84.6) | 20/27  (74.1) | 41.5 |
| ≥ 60 years | 5 (4.5%) | 2/2  (1) | 0.09 | 1 | 1/39  (2.6) | 1/27  (3.7) | 0 |
| Life style |  |  |  |  |  |  |  |
| urban | 79 (71.8%) | 32/53 (0.6) | 0.13 | 11 | 32/39  (82.1) | 21/27  (77.8) | 39.6 |
| rural | 31 (28.2%) | 12/13 (0.92) | 0.12 | 6 | 7/39  (17.9) | 6/27  (22.2) | 7.7 |
| Origin |  |  |  |  |  |  |  |
| Central East Tunisia^a,b^ (Sfax, Gabes) | 44 (40%) | 19/29 (0.64) | 0.13 | 7 | 17/39  (43.6) | 12/27  (44.4) | 34.5 |
| Central west Tunisia^e, f,g^ (Gafsa, Sidi bouzid, Kasserine) | 24 (21.8%) | 8/11 (0.72) | 0.10 | 3 | 6/39  (15.4) | 5/27  (18.5) | 27.3 |
| South east Tunisia^c, d^ (Tataouine, Medenine,) | 38 (34.5%) | 17/26 (0.65) | 0.12 | 7 | 16/39  (41) | 10/27  (37) | 34.6 |
| Sfax^a^ | 23 (21%) | 14/17 (0.83) | 0.14 | 5 | 8/39  (20.5) | 9/27  (33.3) | 17.6 |
| Gabes^b^ | 21 (19%) | 6/12 (0.46) | 0.11 | 3 | 9/39  (23.1) | 3/27  (11.1) | 50 |
| Tataouine^c^ | 23 (21%) | 12/16 (0.75) | 0.11 | 6 | 10/39  (25.6) | 6/27  (22.2) | 25 |
| Medenine^d^ | 15 (13.6%) | 8/10  (0.8) | 0.14 | 4 | 6/39  (15.4) | 4/27  (14.8) | 20 |
| Gafsa^e^ | 13  (11.8%) | 5/6 (0.83) | 0.08 | 3 | 4/39  (10.3) | 2/27  (7.4) | 16.7 |
| Sidi bouzid^f^ | 7 (6.4%) | 3/3 (1) | 0.143 | 2 | 2/39  (5.1) | 1/27  (3.7) | 0 |
| Kasserine^g^ | 4 (3.6%) | 2/2 (1) | 0.16 | 0 | (0) | 2/27  (7.4) | 0 |

γ: P<0.05 (chi-square test)

**(B) Risk factors**

| **Risk factors** | **N°**  **of isolates (%)** | **Genotypic d**  **iversity (Gd^b^)** | **Mean**  **genetic**  **diversity (Hs)** | **N°**  **of clusters** | **N°**  **of clustered isolates (%) *** | **N°**  **of Non-clustered isolates (%)** | **Recent Transmission rate**  **(%)*** |
| --- | --- | --- | --- | --- | --- | --- | --- |
| Raw milk consumption |  |  |  |  |  |  |  |
| yes | 73 (66.4) | 29/44(0.64) | 0.12 | 10 | 25/39  (64.1) | 19/27  (70.4) | 34.1 |
| No | 30 (27.3) | 15/19  (0.8) | 0.13 | 8 | 12/39  (30.8) | 7/27  (25.9) | 21 |
| No data | 7  (6.4) | 3/3  (1) | 0.11 | 2 | 2/39  (5.1) | 1/27  (3.7) | 0 |
| Contact to livestock |  |  |  |  |  |  |  |
| yes | 34 (30.9) | 12/12  (1) | 0.12 | 5 | 5/39  (12.8) | 7/27  (25.9) | 0 |
| No | 53 (48.2) | 22/40  (0.56) | 0.12 | 10 | 28/39  (71.8) | 12/27  (44.4) | 45 |
| No data | 23 (20.9) | 13/14  (0.93) | 0.12 | 6 | 6/39  (15.4) | 8/27  (29.6) | 0 |
| TB history |  |  |  |  |  |  |  |
| yes | 6  (5.4) | 3/3  (1) | 0.17 | 3 | 2/39  (5.1) | 1/27  (3.7) ^γ^ | 0 |
| No | 60 (54.5) | 30/40  (0.75) | 0.13 | 9 | 19/39  (46.2) | 21/27  (77.8) | 25 |
| No data | 44  (40) | 15/23  (0.65) | 0.12 | 10 | 18/39  (46.2) | 5/27  (18.5) | 34.8 |
| BCG vaccination |  |  |  |  |  |  |  |
| yes | 62 (56.4) | 26/38  (0.69) | 0.13 | 9 | 21/39  (53.8) | 17/27  (63) | 31.6 |
| No | 3  (2.7) | 2/2  (1) | 0.12 | 1 | 1/39  (2.6) | 1/27  (3.7) | 0 |
| No data | 45 (40.9) | 18/26  (0.66) | 0.11 | 9 | 17/39  (43.6) | 9/27  (33.3) | 30.8 |

γ: P<0.05 (chi-square test)

*****: Among all the 110 isolates, 66 with major SB (SB0120, SB0121 and SB2025) were used for all analyses done in Table S4 (Libyan isolates and the strains with genotyping missing data were not used).

^a^Gd : number of different genotypes / Total number of isolates in each population
